# Treatment of snoring with positional therapy in patients with positional obstructive sleep apnea syndrome

**DOI:** 10.1038/srep18188

**Published:** 2015-12-11

**Authors:** Wen-Chyuan Chen, Li-Ang Lee, Ning-Hung Chen, Tuan-Jen Fang, Chung-Guei Huang, Wen-Nuan Cheng, Hsueh-Yu Li

**Affiliations:** 1 General Education Center, Chang Gung University of Science and Technology, Tao-Yuan 33303, Taiwan, ROC; 2 Research Center for Industry of Human Ecology, Chang Gung University of Science and Technology, Tao-Yuan 33303, Taiwan, ROC; 3 Department of Otorhinolaryngology - Head and Neck Surgery, Sleep Center, Linkou-Chang Gung Memorial Hospital, Tao-Yuan 33305, Taiwan, ROC; 4 Department of Thoracic Medicine, Sleep Center, Linkou-Chang Gung Memorial Hospital, Tao-Yuan 33305, Taiwan, ROC; 5 Faculty of Medicine, College of Medicine, Chang Gung University, Taoyuan 33303, Taiwan, ROC; 6 Department of Laboratory Medicine, Linkou-Chang Gung Memorial Hospital, Tao-Yuan 33305, Taiwan, ROC; 7 Department of Medical Biotechnology and Laboratory Science, College of Medicine, Chang Gung University, Tao-Yuan 33303, Taiwan, ROC; 8 Graduate Institute of Biomedical Sciences, College of Medicine, Chang Gung University, Tao-Yuan 33303, Taiwan, ROC; 9 Department of Sports Sciences, University of Taipei, Tai-Pei 11153, Taiwan, ROC; 10 Department of Sleep Medicine, Royal Infirmary of Edinburgh, Edinburgh EH16 4SA, UK.

## Abstract

Position therapy plays a role in treating snoring and obstructive sleep apnea syndrome (OSAS). The purpose of this study was to investigate whether position therapy using a head-positioning pillow (HPP) could reduce snoring sounds in patients with mild-to-moderate positional OSAS, taking into account the potential confounding effects of body weight. A total of 25 adults with positional OSAS (apnea-hypopnea index [AHI]_supine_:AHI_non-supine_ ≥ 2) were prospectively enrolled. Patients were asked to use their own pillows at home during the first night (N_0_), and the HPP during the second (N_1_) and third (N_2_) nights. The primary outcome measures included the subjective snoring severity (SS, measured on a visual analogue scale ranging from 0 to 10) and the objective snoring index (SI, expressed as the number of snoring events per hour measured on an acoustic analytical program). Both endpoints were recorded over three consecutive nights. From N_0_ to N_2_, the median SS and SI values in the entire study cohort decreased significantly from 5.0 to 4.0 and from 218.0 events/h to 115.0 events/h, respectively. In the subgroup of overweight patients, SS showed a significant improvement, whereas SI did not. Both SS and SI were found to be significantly improved in normal-weight patients.

Obstructive sleep apnea syndrome (OSAS) is a widespread sleep disorder affecting 2–4% of the middle-aged population^1^. Common symptoms and signs of OSAS include snoring, shallow breathing alternating with apnea, and excessive daytime sleepiness^2^. In the absence of an effective treatment, OSAS can cause sleep deprivation in both snorers and their bed partners^3^. Moreover, mounting evidence suggests that OSAS may increase the risk of adverse cardiovascular events and all-cause mortality^4^. Although continuous positive airway pressure (CPAP) remains the standard treatment for severe OSAS, a recent meta-analysis demonstrated that its use can significantly increase the body mass index (BMI)^5^.

Positional OSAS – defined as an apnea-hypopnea index (AHI) ≥ 5 and an AHI in the supine position (AHI_supine_):AHI in the non-supine position (AHI_nonsupine_) ≥ 2 –6 occurs in more than 50% of OSAS patients^7^. Patients with positional OSAS tend to have less disease severity, lower BMI, younger age, and better outcomes after palatal surgery^8^,^9^. Positional therapy has been proposed as a potentially useful strategy to avoid supine sleeping and consequently reduce the severity of positional OSAS. Specifically, patients with positional OSAS and an AHI_nonsupine_ < 5 are ideal candidates for positional therapy. Several different devices – including positional alarms^10^, tennis balls^11^, elevation pillows^12^, lateral sleep pillows^13^, vests^14^, neck-worn vibration systems^15^, and sleep position trainers^16^ – have been developed for positional therapy. Although a recent meta-analysis demonstrated that positional therapy is inferior to CPAP for reducing the severity of OSAS^17^, its potential usefulness for mild-to-moderate positional OSAS may be under-appreciated^18^. Notably, no direct relationship between the level of OSAS and the subjective severity of snoring exists. Data on the potential clinical utility of positional therapy for reducing snoring remain scanty, and the potential confounding effects of BMI have not been adequately taken into account.

Because the occurrence of pharyngeal collapse and snoring may be dependent on the head position^19^,^20^, we reasoned that a head-positioning pillow (HPP) designed to avoid supine sleep may serve as a simple and effective tool for reducing the severity of snoring in patients with uncomplicated mild-to-moderate positional OSAS^7^,^18^. We therefore designed the current prospective study to investigate the potential efficacy and safety of an *ad-hoc* designed HPP aimed at reducing snoring in uncomplicated patients with mild-to-moderate positional OSAS (AHI_nonsupine_ < 5). Volunteers who were willing to undergo snoring positional therapy who met the inclusion criteria were investigated, and the potential confounding effect of BMI was taken into account.

## Results

### Study population

Of the 32 eligible adult patients, seven were excluded because of the following reasons: pregnancy (*n* = 1), ischemic heart disease (*n* = 1), BMI ≥ 30 kg/m^2^ (*n* = 3), cervical spondylosis (*n* = 1), and untreated depression (*n* = 1). Consequently, a total of 25 patients (median age: 47.0 years, 84% males) were included in the final analysis (Table 1). The median BMI was 24.8 kg/m^2^, and 80% of the study patients complained of moderate-to-severe snoring. Table 1 also summarizes the general characteristics of the study participants according to their BMI. Thirteen patients (52%) were normal-weight, and 12 (48%) were overweight. Positional changes in retroglossal collapsibility of the longitudinal diameter (Col_LD_) in the normal-weight group were found to be statistically significant (supine *versus* lateral: −8.1% *versus* −1.9%, *p* = 0.019; power = 70%).

### HPP safety

**Figure 1:**
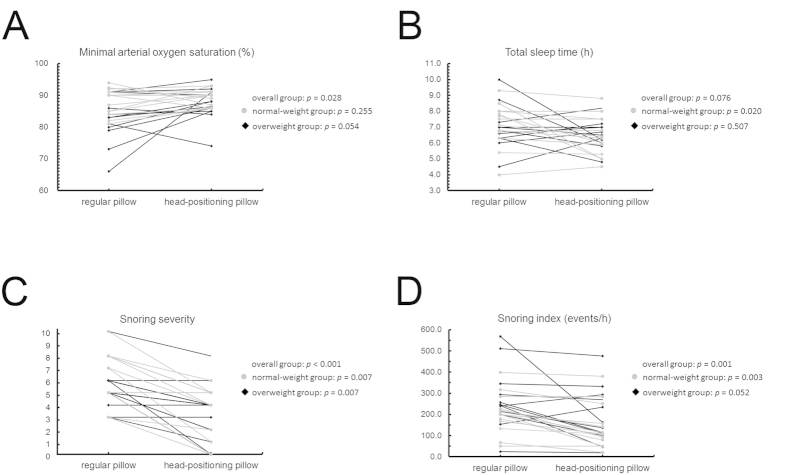
Changes in the main variables of interest induced by the switch from a regular pillow to a head-positioning pillow (HPP). (**A**) In the entire study cohort, the use of a HPP caused a significant increase in the minimal arterial oxygen saturation. (**B**) In normal-weight patients, the use of a HPP resulted in a decreased total sleep time. (**C**) In the entire study cohort, the use of a HPP caused a significant reduction in the severity of snoring. (**D**) Both in the entire study cohort and in normal-weight patients, the use of a HPP results in a significant reduction of the snoring index.

Patients were asked to use their regular pillows at home during the first night (N_0_) and the HPP during the second and third nights (N_1_ and N_2_). Table 2 summarizes the HPP usage data and the differences in total sleep time and arterial oxygen saturation during the use of regular pillow and HPP. Twelve (48%) participants did not show any obvious discomfort (discomfort score, 0–2), whereas 8 (32%) reported a mild discomfort (discomfort score, 3–4). All of the subjects correctly utilized the HPP during N_2_. The median minimal arterial oxygen saturation (SaO_2_) was significantly higher in N_2_ than in N_0_ (88.5% *versus* 85.5%, respectively, *p* = 0.028; power = 71%; Fig. 1, panel A). Discomfort scores associated with the HPP use and the minimal SaO_2_ measured on N_0_ were found to be BMI-dependent. The median total sleep time was significantly higher when subjects slept with their regular pillow as compared with HPP (7.5 h *versus* 6.3 h, respectively, *p* = 0.020; power = 67%; Fig. 1, panel B). However, most of the subjects (85%) reported spontaneous awakening and feeling refreshed after HPP use. No adverse effects were reported by the study participants throughout the entire study period.

### Outcome measures

Table 3 summarizes the primary and secondary outcome measures. Both snoring severity (Fig. 1, panel C) and the snoring index (Fig. 1, panel D) were significantly lower in N_2_ than in N_0_. Specifically, the median snoring severity decreased by 33.3% from an N_0_ value of 5.0 to an N_2_ value of 4.0 (*p* < 0.001; power = 98%). In addition, the median snoring index decreased by 34.4% from an N_0_ value of 218.0 events/h to an N_2_ value of 115.0 events/h (*p* = 0.001; power = 90%). The snoring severity at N_2_ was significantly associated with baseline retroglossal-Col_LD_ (*r* = −0.464, *p* = 0.019) in the supine position. At N_2_, the criterion for the first primary outcome measure (reduction to an N_2_ snoring severity score of 3 or less) was met by 36% of the study participants (*n* = 9; lower boundary of the 97.5% confidence interval [CI], 16). The criterion for the second primary outcome measure (reduction of at least 25% in the snoring index score) was met by 52% of the patients (*n* = 13; lower boundary of the 97.5% CI, 36).

Subgroup analyses in normal-weight patients revealed that both snoring severity (N_0_ *versus* N_2_: 7.0 *versus* 5.0; *p* = 0.007; power = 92%) and snoring index (N_0_ *versus* N_2_: 200.0 *versus* 107.0; *p* = 0.003; power = 94%) were significantly lower when the HPP was used. Similarly, overweight patients had a reduced snoring severity when HPP was used (N_0_ *versus* N_2_: 5.0 *versus* 4.0, *p* = 0.007; power = 86%), although the snoring index was not significantly so (N_0_ *versus* N_2_: 244.0 *versus* 149.5, *p* = 0.052; power = 62%). BMI did not show a dose-dependent effect on the magnitude of reduction of both the snoring severity and the snoring index.

**Figure 2:**
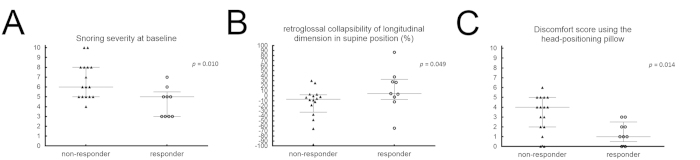
Changes in the main variables of interest in responders and non-responders. (**A**) At baseline, the snoring severity of non-responders was significantly higher than that of responders. (**B**) The retroglossal collapsibility of longitudinal dimension in the supine position in non-responders was significantly lower than that of responders. (**C**) The discomfort score using the head-positioning pillow was significantly higher in non-responders than in responders.

Compared with non-responders, subjective responders had 1) a lower median snoring severity at the baseline visit performed before enrollment (5.0 *versus* 6.0, *p* = 0.010; power = 83%; Fig. 2, panel A), 2) a higher median Col_LD_ of the retroglossal space in the supine position using the HPP (4.0% *versus* −6.8%, *p* = 0.049; power = 54%; Fig. 2, panel B), and 3) a lower median discomfort score during N_2_ (1.0 *versus* 4.0, *p* = 0.014; power = 79%; Fig. 2, panel C; Table 4). We also identified significant correlations between these three factors and subjective responses (*r* = −0.525, 0.404, and −0.497, respectively; *p* = 0.007, 0.045, and 0.012, respectively). Patients who showed an objective response did not differ from other participants in all of the variables of interest.

### Secondary outcomes

Full-night snoring sound analysis did not identify significant differences in mean sound intensity, maximal sound intensity, mean sound frequency, and peak sound frequency (Table 3).

## Discussion

In this study, we investigated the potential usefulness of a HPP for the positional therapy of non-obese adults with positional OSAS, with a special focus on the potential confounding impact of BMI. As expected, the use of a HPP in normal-weight patients resulted in positive subjective and objective outcomes. Changes in the snoring index were not statistically significant in the subgroup of overweight patients, even though they reported a significant improvement in the snoring severity. Another important finding of the current report is the reduction of subjective symptoms in patients who underwent positional therapy, most notably if they had a relatively low snoring severity, a high retroglossal collapsibility in the longitudinal direction while lying supine, and less discomfort with the use of HPP. Despite limited by the small sample size and subject to future confirmation, our findings provide a rationale for BMI-directed positional treatment of snoring in patients with positional OSAS.

The first report on the potential effects of positional therapy on snoring events dates back to 1982^21^. More recently, several studies demonstrated that positional therapy can avoid the patient to assume the supine position while asleep^10^,^11^,^12^,^13^,^14^,^15^,^16^. In this scenario, the use of specifically designed pillows has been proposed as a viable strategy to reduce the number of adverse respiratory events during sleep (without taking positional dependency into consideration) in patients with mild-to-moderate OSAS^12^,^13^. Unfortunately, the low compliance to the use of such devices represents a common cause of therapeutic failure^22^. To overcome these issues, a novel HPP specifically designed (1) to elevate the head and neck during supine sleeping and (2) to allow free lateral sleeping without limitations to the arms was utilized in this study. Notably, the use of HPP in our report was associated with a relatively low discomfort and excellent short-term compliance.

A potential reason that may explain why oxygen saturation did not decrease in parallel with the snoring scores in our study is that HPP could have reduced snoring by waking the patients. A full-night polysomnography monitoring would be required to thoroughly appreciate the effect of HPP on OSAS severity. We are thus planning to investigate the changes in polysomnography variables associated with the use of HPP in OSAS patients in a future randomized, double-blind, placebo-controlled, two-period, cross-over clinical trial. While lying in the supine position, OSAS patients tend to adapt their craniofacial attitude to maintain a patent upper airway (through the extension of the neck and the anterosuperior positioning of the hyoid bone coupled with upward and forward mandible rotation)^23^. In this scenario, the HPP is aimed at maintaining the head and neck in the sniff position during supine sleeping, with the ultimate goal of decreasing upper airway obstruction. Here we show that the use of the HPP increased both the minimal and mean SaO_2_, without significantly affecting the total sleep duration in the entire study cohort. Moreover, awake endoscopy revealed that the need for expansion of the retroglossal space in the longitudinal dimension (aimed at contrasting the forceful inspiratory drive) was significantly reduced by the sniff position in the normal-weight group but not in overweight patients. In addition, the HPP was found to increase the sleep efficiency of normal-weight participants through a decrease in total sleep time and a refreshed awakening. Nonetheless, no significant differences in total sleep time were observed in the overweight group.

Zuberi *et al.*^13^ have previously shown that the use of a supine sleep avoidance pillow resulted in a subjective decrease of snoring in 78% of users. In contrast, Michaelson and Mair^24^ reported that an ergonomically-shaped pillow designed to realign the head and neck did not significantly improve subjective and objective snoring. In the current study, 72% of the study patients reported subjective improvements and 36% responded well to the HPP as determined by a decrease in the snoring severity to an absent-to-mild level. Taken together, our results support the clinical utility of the short-term HPP use for reducing the sleep disturbances associated with snoring.

An important finding of this study is the inverse relation between the reduction in snoring severity elicited by the use of a HPP and the retroglossal-Col_LD_ measured using awake nasopharyngoscopy with the Müeller’s maneuver while lying in the supine position. It is thus conceivable that retroglossal-Col_LD_ could play a major role in determining the response to HPP-based positional therapy. In general, the treatment of non-severe positional OSAS should be aimed at overcoming the increased Col_LD_ of the retroglossal airway. We hypothesize that the HPP could exert its beneficial effects on snoring severity by efficiently stretching the lateral pharyngeal wall when the patient lies in the sniff position while asleep. More importantly, the use of HPP successfully prevented the snoring resulting from supine sleeping. Notably, the stabilization of both the retroglossal-Col_LD_ and Col_TD_ measures elicited by the HPP was more evident when the patient was asleep in the lateral position as compared with waking consciousness.

Overweight patients did not show the statistically significant decrease in the snoring index observed in the normal-weight group. The exact mechanisms through which BMI could affect the response to positional therapy in positional OSAS remain unclear. In general, OSAS patients tend to have an abnormally thick lateral pharyngeal wall that may cause the obstruction of the upper airway^25^. Moreover, patients with positional OSAS are characterized by wider lateral airways (because of a thinner lateral pharyngeal wall), lower facial height, and a more pronounced backward position of the lower jaw^26^. Notably, we observed that overweight participants tended to have a low longitudinal collapsibility of the retroglossal airway in the supine position. Differently from the findings obtained in normal-weight subjects, we noted that the HPP use was unable to modify the longitudinal collapsibility at this level in overweight participants (Table 1). We thus speculate that the HPP used in this study was unable to counterbalance the collapse of the pharyngeal space, the frequency of palatal fluttering, and the gravitational forces associated with snoring in the subgroup of overweight patients.

Several limitations of our study merit comment. Although the severity of snoring was used as the primary outcome measure, snoring-related quality of life was not assessed. Current questionnaires aimed at investigating the quality of life calculate average scores over longer time periods, ultimately being unsuitable for short-term studies. The baseline nasopharyngoscopy was performed during waking consciousness only, and an accurate investigation of the upper airway during sleep was not performed. The use of different pillows (normal pillows *versus* HPP) was not blinded, and we cannot exclude that the subjective improvement could be attributed at least in part to a learning effect. Moreover, we did not specifically analyze the data collected on the second night (N_1_) to minimize the potential overlapping pillow effects^17^. In this scenario, a 7-day learning period before the use of a new pillow is a potentially useful strategy to minimize the psychological effects occurring during the first study days. Importantly, caution should be exercised in the generalization of our data. Accordingly, the sample size was relatively small and only short-term outcomes were investigated.

The majority of OSAS patients attending our sleep clinics have severe manifestations and are at a high risk of developing cardiovascular and metabolic complications. The clinical management of these patients requires an aggressive treatment and tight follow-up protocols. However, conservative strategies (including positional therapy) remain a feasible option for patients with mild-to-moderate OSAS. The latter population is deemed to be at low cardiovascular risk and was the focus of the current study. Although subject to further confirmation, we believe that the use of the HPP may be considered as a viable therapeutic option as an initial treatment for adult patients (1) who require treatment of primary snoring, (2) have positional OSAS with an AHI_non-supine_ < 5, and (3) are not overweight. These criteria are slightly more restrictive than those outlined in the clinical practice guidelines for the treatment of OSAS with oral appliances (approved in 2015 by the American Academy of Sleep Medicine and American Academy of Dental Sleep Medicine)^27^. Such guidelines recommend the use of oral appliances (rather than no therapy) for adult OSAS patients who request treatment of primary snoring and are intolerant to CPAP therapy (or prefer an alternative therapy). Our results emphasize the clinical relevance of both positional dependence and BMI in the positional therapy of snoring.

In conclusion, the results of our short-term study demonstrated that the use of a HPP significantly reduced the subjective and objective severity of snoring in adult patients with mild-to-moderate positional OSAS, with an acceptable safety profile. Positional therapy was associated with subjective improvements of snoring regardless of BMI. However, overweight patients did not show an objective reduction of the snoring index, suggesting the potential usefulness of a weight reduction strategy to ameliorate their outcomes. Before the widespread clinical use of HPP can be recommended, well-designed, long-term clinical trials are needed to confirm and expand our pilot findings. In this scenario, the current data should not be intended as a basis for guiding treatment decisions.

## Methods

### Ethics statement

This study was designed as a prospective case series. Ethics approval was granted by the Institutional Review Board of the Linkou-Chang Gung Memorial Hospital, Taoyuan, Taiwan (102-0064A3). All procedures were in compliance with the current regulations. Written informed consent was obtained for all participants.

### Study population

Between January 1, 2013 and December 31, 2013, we recruited from our otolaryngology clinics a total of 32 consecutive adult patients aged between 20 and 65 years who presented with polysomnography-confirmed, mild-to-moderate (5 ≤ AHI < 30) positional OSAS. All participants expressed subjective complaints during the previous six months. Moreover, they reported during the baseline visit a significant reduction of their snoring while lying in the non-supine position. All participants were willing to receive positional therapy with the use of the HPP for snoring reduction. Patients were excluded either in presence of (1) marked anatomical abnormalities (e.g., tonsillar hypertrophy or tongue obstruction) that prevented the effective use of the HPP or (2) a complete concentric collapse of the retropalatal airway on nasopharyngolaryngoscopy performed with the Müeller’s maneuver. Additional exclusion criteria were as follows: (1) AHI_non-supine_ ≥ 5; (2) pregnancy or breastfeeding, (3) recent history of cardiovascular disease, stroke, or pulmonary disorders, (4) BMI ≥ 30 kg/m^2^, (5) spine disorders hindering the lateral sleep position, (6) insomnia, dementia, or psychiatric disorders, and (7) unwillingness to participate in the study. Demographic data, BMI, clinical symptoms, and the results of physical examinations were collected in all participants. BMI was defined as the weight in kilograms divided by the square of the height in meters (kg/m^2^). According to their BMI, the study subjects were divided into normal-weight (BMI, 18.5–24.9 kg/m^2^) and overweight (BMI, 25.0–29.9 kg/m^2^) patients^28^. The collapsibility of the retropalatal and retroglossal spaces was assessed using a flexible nasopharyngoscope (video rhinolaryngoscope, ENF Type V2; digital processor, VISERA OTV-S7; light source, VISERA CLV-S40; Olympus Optical Co., Ltd., Tokyo, Japan) associated with a picture archiving and communication system (Centricity Enterprise Web V3.0.10; GE Healthcare, Chalfont, UK). The system allowed a direct measure of spatial dimensions using a length measuring tool. Subjects utilized the HPP either in the supine or in the lateral position and underwent transnasal nasopharyngoscopy using the Müeller’s maneuver. Col_TD_ (%) and Col_LD_ (%) values were obtained in all participants^29^.

### Polysomnography

Polysomnography recordings were performed in a laboratory setting under the supervision of expert technicians. The polygraph system (Nicolet UltraSom System, Madison, WI, USA) used for the study consisted of an electroencephalogram, a submental and anterior tibialis electromyogram, an electro-oculogram, and an electrocardiogram. The airflow was monitored using a nasal pressure transducer, whereas respiratory inductive plethysmography was used to assess thoracoabdominal motion. The body position during sleep (e.g., left, right, prone, and supine) was determined with a position sensor (Compumedics, Abbotsford, Victoria, Australia) attached by Velcro^®^ straps to the respiratory band midline over the sternum. Before recordings, the body position sensor was calibrated by moving the patient through various simulated sleep positions. During polysomnography, the study patients were advised to spend >5% of the total sleep time either in supine or in lateral position. However, they were asked to sleep as they usually do at home as much as possible^30^. All of the signals were digitally recorded, sampled, filtered, and stored using a sample rate up to 64 Hz. The recordings took place between 22:00 p.m. and 06:00 a.m. Sleep stages were manually scored according to standard criteria established by the American Academy of Sleep Medicine (2007 version)^2^. Apneas were defined as pauses in breathing of more than 10 sec while asleep. Hypopneas were scored when the peak signal excursions dropped by ≥30% of pre-event baseline using nasal pressure for ≥10 sec in association with either ≥ 4% arterial oxygen desaturation or an arousal. The AHI was calculated as the number of total apnea and hypopnea episodes per hour of sleep. AHI_total_, AHI_supine_, AHI_nonsupine_, mean SaO_2_, and minimal SaO_2_ were recorded in all participants. Positional OSAS was defined as an AHI_supine_ at least twice of the AHI_nonsupine_ if the patient spent more than 5% of the total sleep time in either the supine or the non-supine position^30^. Otherwise, the subject was considered to have a non-positional OSAS and excluded from the initial survey.

### HPP

**Figure 3:**
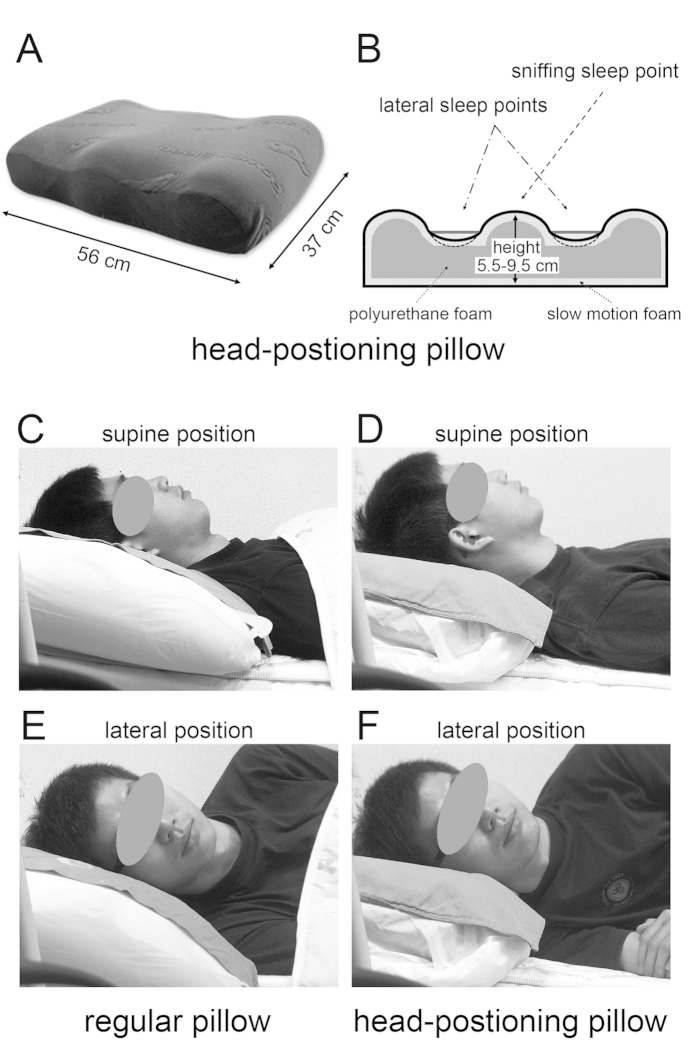
Photographs of a 26-year-old normal-weight (body mass index, 21.5 kg/m2) male patient with moderate obstructive sleep apnea syndrome (apnea-hypopnea index, 15.9 events/h) using either a regular pillow or the head-positioning pillow (HPP). (**A**) Original image of the HPP pillow. (**B**) Cross-sectional image and materials used for fabricating the HPP pillow. (**C**) The median portion of a regular pillow supports the head and neck in the usual position. (**D**) The median portion of the HPP is the highest point that can help keeping the head and neck in the sniff position and maintaining the patent airway in the lateral position. (**E**) The bilateral paramedian portions of a regular pillow have the same height as the median portion and provide no additional benefits during lateral sleep. (**F**) The bilateral paramedian portions of the HPP are its lowest parts; such design can ultimately promote head and neck rotation to the lateral sleep position and maintain the patent airway.

**Figure 4:**
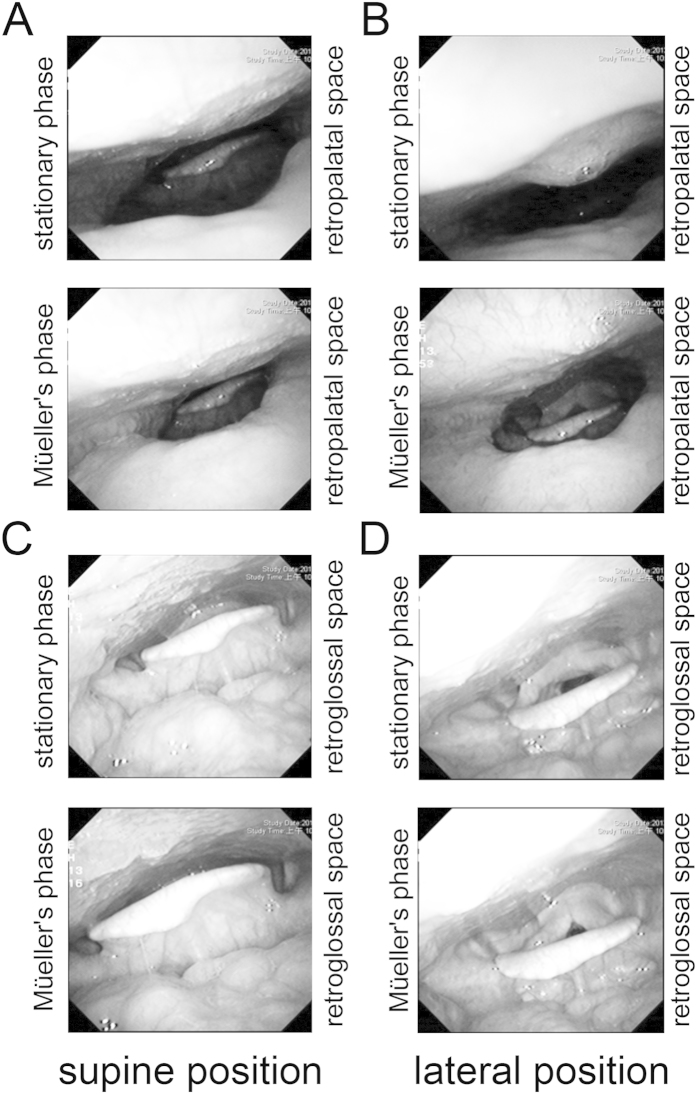
Nasopharyngolaryngoscopy findings of the same patient reported in Figure 3 (snoring severity, 7; snoring index, 399.0 events/h) in different sleep positions. (**A**) While in supine (sniff) position, the patient’s retropalatal space easily collapsed from the stationary phase to the Müeller’s phase. (**B**) While in lateral position, the retropalatal space was relatively stable between the two phases. (**C**) While in supine (sniff) position, the retroglossal space collapsed in a relatively easily manner from the stationary phase to the Müeller’s phase. (**D**) While in lateral position, the retroglossal space was widened and stable between the two phases.

The study participants were required to use their regular pillow and the HPP (Power Sleep^®^ anti-snore pillow [Taiwan Patent Number M446586], Green-Sweet Mattress Corp., New Taipei City, Taiwan) both at our clinics and at home (Fig. 3, panel A; dimensions: 56-cm length × 37-cm width × 5.5~9.5-cm height). According to the manufacturer’s specifications, the integrated HPP consists of an inner layer (made of firm polyurethane foam with a hardness of 53 kg/314 cm^2^) and a superficial layer (consisting of a 2.5-cm-thick slow-motion polyurethane foam with a hardness of 10 kg/314 cm^2^; Fig. 3, panel B). The HPP can be routinely cleaned using a common sponge. Its median portion is strengthened with firm polyurethane foam, ultimately allowing an adequate support of the head and neck for any subject with a body weight of 110 kg or less without undergoing significant deformations. Because the HPP median portion is narrower and firmer than the lateral sleep parts, its use tends to promote head turning to the lateral sleep position. Consequently, subjects tend to spend more time in the lateral sleep position than in the sniff position. The HPP is available with three different heights of the median portion (small, 5.5 cm; medium, 7.5 cm; large, 9.5 cm). The optimal HPP height was selected based on the widest retroglossal space achieved during flexible nasopharyngoscope examination. Such an optimal height can maintain the head and neck in the sniff position (extension of the head and flexion of the neck over the trunk)^31^ in order to straightly align the upper respiratory tract and widely open the upper airway (Fig. 4, panels A/C) during supine sleep on the HPP median portion (Fig. 3, panels B/D). When the head moves from the highest median portion to the lowest lateral portion of the HPP (Fig. 3, panel B), the lateral rotation of the head (lateral sleep; Fig. 3, panel F) may decrease the collapsibility of the upper airway (Fig. 4, panel B), ultimately preventing tongue dropping (Fig. 4, panel D). Theoretically, both head positions can maintain a patent airway and reduce snoring sounds.

The safety of the HPP was assessed using the following three parameters: 1) a discomfort score calculated on a visual analogue scale (VAS) ranging from 0 (no discomfort) to 10 (markedly severe discomfort preventing HPP use), 2) compliance to the device use (HPP usage time/total sleep time × 100 [%]), and 3) total sleep time. A portable wrist pulse oximeter (3100 WristOx, Nonin Medical, Inc., Minneapolis, MN, USA) was used for continuous recording of SaO_2_ and identification of arterial oxygen desaturation (greater than 4% decrease from baseline SaO_2_) for at least 6 h sleep time on N_0_, N_1_, and N_2_.

### Subjective assessment of snoring sounds

Subjective assessment of snoring was performed based on the bed partner’s description of symptoms using a VAS questionnaire ranging from 0 (no snoring) to 10 (markedly severe snoring, bed partner leaves the bedroom)^32^,^33^. All of the study participants’ bed partners were interviewed to investigate the severity of snoring during the three recording nights (either with the use of a regular pillow or HPP). They were also asked whether the patient snored differently while asleep using the HPP in the non-supine position during the first week after the study.

### Objective assessment of snoring sounds

Objective analysis of snoring sounds was performed through the full-night recording of snoring sounds via a snore detection system. Because the in-door environments in Taiwan are characterized by certain spatial limitations (e.g., an 8-foot-high ceiling), we carefully removed from acoustic analysis all of the high frequency sounds (>3000 Hz) considered as the results of wall and/or ceiling reflections. To account for the effects of acoustic environment, snoring sounds were input via a linear pulse code modulation portable digital recorder (PCM-D50, Sony Electronics Inc., Tokyo, Japan) equipped with two built-in high-performance unidirectional dynamic microphones. The recorder was vertically positioned 100 cm above the patient’s head^32^. When the bed headboard was attached to a wall or a corner, the participants were asked to maintain a minimum distance from the board (>1/4 loading space). Snoring sounds in the frequency range from 40 Hz to 2000 Hz were analyzed as described previously^32^,^34^. Using high-fidelity loudspeakers, we have previously shown slight differences (±3 dB) in this frequency band when reproducing snoring sounds recorded at home *versus* the sleep laboratory^20^. All subjects were asked to enter their bedroom quietly (background noise <30 dB) and sleep alone. The background bedroom sounds were recorded for 10 min. After falling asleep spontaneously, continuous recordings of the participants’ sleep sounds were performed for at least 6 h at a sampling rate of 44,100 Hz. We used the fast Fourier transformation (range: 20–2,000 Hz) for the estimation of the frequency power spectra. Snoring episodes were subsequently identified using a validated automatic analyzer developed by our research group (Snore Map, Chang Gung Memorial Hospital, Taoyuan, Taiwan)^32^,^33^,^35^. The highest intensity of background noises at the beginning of each test was found to be relatively constant in the frequency range from 20 Hz to 40 Hz. The interference of background noises on snoring sounds was reduced using a high-pass filtering technique. All-night analysis of snoring signals was performed using an automatic detection algorithm based on the following two criteria: (1) sound energy >0.05 au and (2) sound duration between 0.6 sec and 4.0 sec. The snoring sound detection algorithm was developed on an adaptive energy threshold^31^. Using this methodology, the following parameters were calculated: (1) snoring index (events/h), (2) mean sound intensity (dB), (3) maximal sound intensity (dB), (4) mean sound frequency (Hz), and (5) peak sound frequency (Hz) in the 40−2,000 Hz domain.

### Outcome measures

The primary outcome measure was the change in snoring sounds (as assessed by the medians of the subjective snoring severity and the objective snoring index) from baseline (N_0_) to the second night (N_2_). We also considered as a primary outcome measure the proportion of participants in whom the use of HPP induced a significant response from N_0_ to N_2_ (based on the snoring severity and the snoring index). The response to the use of HPP was considered significant if the N_2_-snoring severity score was ≤ 3^36^. Because the HPP cannot modify the anatomical structure of the upper airway, we assessed its impact on the frequency of the snoring events (snoring index determined by acoustic analysis). Patients with OSAS spend ~75% of their total sleep time in the supine position, with the remaining 25% being in the non-supine position^30^. In general, most studies have defined good adherence as the use of a sleep device for a minimum of 4 h per night^37^. Accordingly, we assumed that the use of the HPP would result in (1) a snoring index_supine_:snoring index_non-supine_ ratio ≥ 2, and (2) the maintenance of head and neck in the non-supine position for at least 90% of the usage time during ordinary sleep at home. The snoring index associated with the use of the regular pillow was then estimated as follows:

6 h-snoring index_regular pillow_ = 75% × snoring index_supine_ + 25% × snoring index_non-supine_ = 75% × 2 × snoring index_non-supine_ + 25% × snoring index_non-supine_ = 175% × snoring index_non-supine_.

The snoring index associated with the use of the HPP for 4 h and the regular pillow for 2 h was calculated as follows:

4 h-snoring index_HPP_ + 2 h-Snoring index_regular pillow_ = [4 h × (10% × snoring index_supine_ + 90% × snoring index_non-supine_) + 2 h × (75% × snoring index_supine_ + 25% × snoring index_non-supine_)]/6 h = [4 × (110% × snoring index_non-supine_ + 2 × 175% × snoring index_non-supine_]/6 = 132% × snoring index_non-supine_

We estimated that the use of the HPP for at least 4 h per night would reduce the snoring index by at least 24.6% from baseline values (under the assumption of a reduced rate of snoring index = [175% × snoring index_non-supine_ – 132% × snoring index_non-supine_]/[175% × snoring index_non-supine_] × 100%). The response to the use of HPP was therefore considered significant in presence of a ≥25% reduction in the snoring index from baseline values. The secondary outcome measures included the mean sound intensity, the maximal sound intensity, the mean sound frequency, and the peak sound frequency.

### Statistical power

The sample size required for the study was estimated using the primary outcome effects (changes in snoring severity and in the snoring index) observed in a pilot study of three volunteers who had residual positional OSAS following relocation pharyngoplasty. Their snoring severity was 10, 8, and 6, respectively, on night N_0,_ and 6, 4, and 4, respectively, on night N_2_. Their snoring index was 67.0, 40.0, and 154.0 events/h, respectively, on night N_0,_ and 27.0, 22.0, and 84.0 event/h, respectively, on night N_2_. Using an effect size of 1.549 [0.284], a type I error of 0.05, and a 95% power, at least 8 patients were required for identifying statistically significant differences in terms of snoring severity (two-tailed Wilcoxon signed-rank test). However, the minimum sample size to ensure the detection of differences in the snoring index consisted of 23 patients (two-tailed Wilcoxon signed-rank test). Considering a drop-out rate of 20%, a total of 32 consecutive patients were conservatively included in the study.

### Statistical analysis

Because of the small sample size, all variables were analyzed using a non-parametric approach. Continuous data are summarized as medians with interquartile ranges, whereas categorical data are presented as counts and percentages. To avoid the confounding effect related to the first study night, comparisons were made between N_0_ and N_2_ sleep data. The percent (%) change ([N_2_ (or HPP) value − N_0_ (or regular pillow) value]/[N_0_ (regular pillow) value] × 100) was calculated for the variables of interest. Pairwise comparisons were made using the Wilcoxon signed-rank test. The Mann-Whitney *U* test was used for independent variables. The statistical power was calculated for each outcome measure using a two-tailed Wilcoxon signed-rank test or Mann-Whitney *U* test (parent distribution, normal; *β*/*α* ratio, 4). Categorical variables were analyzed with the Fisher’s exact test or the χ^2^ test, as appropriate. Correlations between variables were tested using the Spearman’s rank correlation coefficient. Statistical calculations were performed using the G*Power (version 3.1.9.2; University Kiel, Germany), IBM SPSS (version 22; IBM, Armonk, NY, USA), and the GraphPad Prism for Windows (version 6.01; GraphPad Inc., San Diego, CA, USA) software packages. Two-tailed *p* values < 0.05 were considered statistically significant.

## Additional Information

**How to cite this article**: Chen, W.-C. *et al.* Treatment of snoring with positional therapy in patients with positional obstructive sleep apnea syndrome. *Sci. Rep.* **5**, 18188; doi: 10.1038/srep18188 (2015).

## Figures and Tables

**Table 1:** General characteristics of the entire study cohort and according to the patients’ body mass index.

	**Entire cohort (*****n***** = 25)**	**Weight status**		
		**Normal-weight (*****n***** = 13)**	**Overweight (*****n***** = 12)**	***p*** **value**
Body mass index (kg/m^2^)	24.8 (23.1, 26.4)	23.2 (22.4, 23.8)	26.4 (25.8, 27.5)	<0.001
Age, years	47.0 (32.0, 53.0)	50.0 (31.0, 57.0)	42.5 (36.3, 52.0)	0.650
Sex (male, %)	21 (84)	11 (85)	10 (83)	1.000
Neck circumference (cm)	38.0 (36.0, 40.0)	37.0 (33.0, 38.8)	40.0 (37.1, 40.0)	0.022
Snoring severity	5.0 (5.0, 7.5)	7.0 (5.0, 8.0)	5.0 (4.3, 6.0)	0.247
S-Retropalatal-Col_TD_ (%)^A^	27.0 (13.6, 51.2)	18.8 (11.7, 43.1)	32.6 (16.1, 52.9)	0.437
S-Retropalatal-Col_LD_ (%)^A^	28.3 (11.1, 51.1)	28.3 (11.4, 50.3)	29.6 (−6.8, 59.4)	0.769
S-Retroglossal-Col_TD_ (%)^A^	14.2 (6.9, 23.4)	12.8 (4.1, 25.1)	15.7 (12.4, 24.4)	0.406
S-Retroglossal-Col_LD_ (%)^A^	−2.3 (−15.2, 15.1)	−8.1 (−50.9, 1.5)^B^	3.7 (−7.8, 29.6)	0.052
L-Retropalatal-Col_TD_ (%)^A^	37.6 (20.6, 466.9)	31.1 (14.0, 46.8)	40.3 (26.2, 49.3)	0.205
L-Retropalatal-Col_LD_ (%)^A^	23.6 (−4.2, 52.6)	20.9 (−12.9, 44.7)	34.1 (−3.2, 54.1)	0.376
L-Retroglossal-Col_TD_ (%)^A^	9.6 (6.9, 20.2)	9.1 (8.0, 17.5)	17.6 (4.7, 33.4)	0.538
L-Retroglossal-Col_LD_ (%)^A^	−0.8 (−21.0, 10.5)	−1.9 (−21.9, 12.3)^B^	−0.6 (−3.3, 12.5)	0.611
AHI_total_, events/h	7.0 (6.0, 15.2)	7.0 (6.4, 13.8)	7.0 (5.5, 17.8)	0.852
AHI_supine_, events/h	10.1 (6.9, 22.0)	10.1 (7.6, 27.2)	10.4 (5.8, 21.7)	0.650
AHI_nonsupine_, events/h	2.2 (0.9, 3.7)	2.0 (0.9, 2.9)	2.4 (0.5, 7.2)	0.611
AHI_supine_:AHI_nonsupine_ ratio	4.0 (2.6, 18.4)	8.7 (2.7, 18.6)	3.0 (2.5, 17.4)	0.470
Mean SaO_2_, %	95.0 (94.0, 96.0)	95.0 (94.5, 95.5)	95.0 (94.0, 96.0)	0.810
Minimal SaO_2_, %	87.0 (85.0, 89.0)	87.0 (86.0, 89.5)	85.0 (82.8, 88.5)	0.137
ODI (4%), events/h	6.6 (3.3, 11.2)	6.6 (3.3, 9.9)	6.8 (2.8, 17.6)	0.538

Note: Continuous data are expressed as medians and interquartile ranges, whereas categorical data are given as counts and percentages. Significant differences are marked in bold. AHI, apnea-hypopnea index; Col_LD_, collapsibility of longitudinal dimension; Col_TD_, collapsibility of transverse dimension; L, lateral position; ODI, oxygen desaturation index; S, supine position; SaO_2_, arterial oxygen saturation.

^A^Collapsibility was measured in the supine position using the head-positioning pillow.

^B^*p* < 0.05 supine position *versus* lateral position using the HPP.

**Table 2:** Differences in airway collapsibility, total sleep time, and arterial oxygen saturation associated with the use of a regular pillow *versus* a head-positioning pillow in the entire study cohort and according to the patients’ body mass index.

	**Overall (*****n***** = 25)**		**Normal-weight (*****n***** = 13)**			**Overweight (*****n***** = 12)**			
	**RP (N**_**0**_)	**HPP (N**_**2**_)	***p*** **value**	**RP (N**_**0**_)	**HPP (N**_**2**_)	***p*** **value**	**RP (N**_**0**_)	**HPP (N**_**2**_)	***p*** **value**
Discomfort score	Reference	3.0 (1.0, 4.0)	NA	Reference	5.0 (2.0, 5.0)^B^	NA	Reference	2.0 (0, 3.0)^B^	NA
Compliance, %	Reference	100 (100, 100)	NA	Reference	100 (100, 100)	NA	Reference	100 (100, 100)	NA
Total sleep time, h	7.1 (6.4, 8.0)	6.4 (5.8, 7.4)	0.076	7.5 (6.5, 8.0)	6.3 (5.2, 7.5)	**0.020**	7.0 (6.3, 7.5)	6.5 (6.0, 7.0)	0.507
Mean SaO_2_, %	95.8 (95.1, 96.1)	96.1 (95.2, 96.8)	0.742	95.5 (65.0, 96.4)	95.7 (94.9, 96.9)	0.861	95.9 (95.7, 96.1)	96.1 (95.6, 96.8)	0.327
Minimal SaO_2_, %	85.5 (82.3, 91.0)	88.5 (86.0, 91.0)	**0.028**	90.0 (84.5, 91.5)^C^	90.0 (86.0, 91.2)	0.255	83.0 (79.0, 91.0)^C^	88.0 (85.0, 91.0)	0.054
ODI, events/h	4.2 (1.5, 8.9)	3.5 (1.6, 8.5)	0.247	4.0 (1.3, 7.7)	3.4 (1.0, 9.1)	0.366	4.4 (2.7, 9.1)	4.3 (2.3, 8.6)	0.346

Note: Continuous data are expressed as medians and interquartile ranges. Significant p values are marked in bold.

HPP, head-positioning pillow; NA, not available; ODI, oxygen desaturation index; RP, regular pillow; SaO_2_, arterial oxygen saturation.

^A^Collapsibility was measured in the supine position.

^B^*p* < 0.05 normal-weight patients versus overweight patients using the HPP.

^C^*p* < 0.05 normal-weight patients versus overweight patients using the RP.

**Table 3:** Changes in snoring sounds induced by the use of the head-positioning pillow in the entire study cohort and according to the patients’ body mass index.

	**Overall (*****n***** = 25)**			**Normal-weight (*****n***** = 13)**			**Overweight (*****n***** = 12)**		
	**RP (N**_**0**_)	**HPP (N**_**2**_)	***p*** **value**	**RP (N**_**0**_)	**HPP (N**_**2**_)	***p*** **value**	**RP (N**_**0**_)	**HPP (N**_**2**_)	***p*** **value**
Primary outcomes									
Snoring severity	5.0 (5.0, 7.5)	4.0 (1.5, 5.0)	** < 0.001**	7.0 (5.0, 8.0)	5.0 (1.5, 5.0)	**0.007**	5.0 (4.3, 6.0)	4.0 (1.3, 4.0)	**0.007**
Snoring index, events/h	218.0 (100.0, 288.5)	115.0 (48.0, 260.3)	**0.001**	200.0 (58.5, 256.0)	107.0 (35.0, 204.3)	**0.003**	244.0 (169.3, 332.0)	149.5 (62.5, 288.0)	0.052
Secondary outcomes									
Mean sound intensity, dB	74.2 (68.4, 81.1)	74.3 (66.7, 80.4)	0.977	72.1 (68.3, 81.0)	71.0 (63.8, 79.4)	0.480	75.8 (68.5, 81.7)	76.0 (70.2, 83.2)	0.583
Maximal sound intensity, dB	90.0 (87.7, 94.2)	90.1 (87.5, 94.2)	1.000	89.8 (89.3, 94.2)	89.0 (84.0, 94.0)	0.308	91.9 (86.1, 96.3)	92.2 (89.6, 94.5)	0.272
Mean sound frequency, Hz	158.4 (113.7, 213.7)	160.2 (129.5, 249.0)	0.549	146.1 (156.6, 194.7)	156.4 (121.3, 218.9)	0.084	185.0 (122.5, 249.2)	167.2 (137.8, 249.4)	0.272
Peak sound frequency, Hz	850.0 (575.0, 1150.0)	810.0 (560.0, 1180.0)	0.939	870.0 (650.0, 1150.0)	715.0 (475.0, 1235.0)	0.656	810.0 (490.0, 1155.0)	825.0 (607.5, 1182.5)	0.556

Note: Continuous data are expressed as medians and interquartile ranges. Significant p values are marked in bold.

HPP, head-positioning pillow; RP, regular pillow.

**Table 4:** Clinical variables in respondent and non-respondents to the use of the head-positioning pillow.

	**Subjective snoring severity**			**Objective snoring index**		
	**No response (*n* = 16)**	**Response (*n* = 9)**	*p* value	**No response (*n* = 12)**	**Response (*n* = 13)**	*p* value
Age, years	46.0 (32.0, 52.5)	49.0 (32.0, 55.5)	0.890	43.5 (33.0, 52.5)	49.0 (31.5, 54.5)	0.689
Sex (male, %)	14 (88)	7 (78)	0.602	10 (83)	11 (85)	1.000
Body mass index, kg/m^2^	24.6 (23.2, 26.4)	25.2 (21.8, 26.6)	0.846	24.8 (22.6, 26.2)	24.0 (23.2, 26.3)	0.769
Neck circumference, cm	37.9 (36.0, 40.0)	38.0 (32.5, 40.0)	0.718	38.8 (36.0, 40.0)	37.8 (35.0, 39.8)	0.574
Snoring severity	6.0 (5.0, 8.0)	5.0 (3.0, 5.5)	**0.010**	5.0 (4.0, 8.0)	6.0 (5.0, 7.5)	0.728
SOS	41.9 (37.6, 51.5)	55.0 (42.1, 58.0)	0.108	43.8 (39.6, 58.0)	47.1 (38.1, 58.0)	0.689
ESS	9.0 (5.0,12.0)	10.0 (6.5, 13.0)	0.598	8.0 (5.0, 12.0)	10.0 (7.0, 12.5)	0.347
Tonsil size	1.0 (1.0, 2.0)	1.0 (1.0, 1.5)	1.000	1.0 (1.0, 2.0)	1.0 (1.0, 1.0)	0.137
FPP	2.0 (1.0, 2.0)	2.0 (1.5, 2.5)	0.452	2.0 (1.0, 2.0)	2.0 (1.0, 2.0)	1.000
S-Retropalatal-Col_TD_ (%)^A^	31.4 (16.7, 52.2)	14.4 (10.2, 51.3)	0.276	32.4 (15.5, 53.4)	17.0 (11.7, 51.2)	0.437
S-Retropalatal-Col_LD_ (%)^A^	34.2 (11.4, 63.2)	20.3 (−0.2, 41.9)	0.452	24.5 (4.2, 42.5)	28.3 (17.4, 67.5)	0.347
S-Retroglossal-Col_TD_ (%)^A^	15.5 (5.9, 29.2)	12.9 (7.8, 19.2)	0.637	14.5 (12.4. 39.8)	14.2 (2.0, 21.3)	0.225
S-Retroglossal-Col_LD_ (%)^A^	−6.8 (−32.5, 2.1)	4.0 (−9.5, 27.8)	**0.049**	3.0 (−7.4, 21.3)	−8.7 (−50.9, 12.8)	0.087
L-Retropalatal-Col_TD_ (%)^A^	36.4 (15.6, 47.3)	37.6 (23.7, 49.3)	0.718	39.3 (23.4, 49.2)	33.3 (17.7, 46.9)	0.769
L-Retropalatal-Col_LD_ (%)^A^	32.9 (8.9, 56.1)	1.0 (−19.2, 35.5)	0.074	6.8 (−15.2, 47.8)	29.9 (10.3, 55.7)	0.295
L-Retroglossal-Col_TD_ (%)^A^	9.3 (7.9, 20.4)	15.5 (5.1, 27.4)	0.890	15.2 (8.5, 31.3)	8.4 (4.2, 19.1)	0.186
L-Retroglossal-Col_LD_ (%)^A^	−2.5 (−21.3, 0.9)	5.7 (−12.6, 28.0)	0.187	−1.6 (−21.7, 23.8)	−0.5 (−18.9, 5.0)	1.000
AHI_total_, events/h	6.8 (5.5, 13.5)	7.4 (5.2, 17.0)	0.890	7.0 (5.8, 8.5)	7.0 (5.2, 15.6)	0.936
AHI_supine_: AHI_nonsupine_ ratio	7.0 (2.7, 18.4)	2.7 (2.2, 16.5)	0.229	2.7 (2.3, 18.4)	10.1 (3.2, 19.6)	0.098
ODI, events/h	6.3 (3.6, 10.5)	6.8 (2.6, 12.1)	0.718	5.9 (4.0 m 7.6)	7.8 (2.6, 11.1)	0.979
N_2__Discomfort score	4.0 (2.0, 5.0)	1.0 (0.5, 2.5)	**0.014**	2.0 (1.0, 4.0)	3.0 (2.0, 4.5)	0.331
N_2__Total sleep time, h	6.6 (5.4, 7.5)	6.3 (5.9, 6.7)	0.677	6.3 (5.3, 6.8)	6.4 (5.9, 7.5)	0.852

Note: Continuous data are expressed as medians and interquartile ranges, whereas categorical data are given as counts and percentages. AHI, apnea-hypopnea index; Col_LD_, collapsibility of longitudinal dimension; Col_TD_, collapsibility of transverse dimension; ESS, Epworth Sleepiness Scale; FPP, Friedman palatal position; L, lateral position; ODI, oxygen desaturation index; S, supine position; SOS, Snoring Outcomes Survey.

^A^Collapsibility was measured in the supine position using the head-positioning pillow.
